# 

**Published:** 1902-03-15

**Authors:** 


### CONTENTS.

Will a High Preliminary Educational Requirement remove the Existing Evil?

*By G. S. Junkerman, M.D., D.D.S.* 109

Dental Education, - - *By Dr. Joseph Griffiths* 117

A Method of Preparing and Filling a Mesio-occlusal Cavity in an Upper Second Bicuspid,

*By Dr. E. K. Wedelstaedt* 131

A Statistical Study of Local Anesthesia in Dentistry,

*By Morris I. Schamberg, D.D.S., M.D.* 137

Is it Worth While to Try to Save the Teeth?

*By Samuel A. Hopkins, M.D , D.D.S.* 143

Care of the Deciduous Teeth,

(*Extracts from a Discussion*) 145

Taking Impression and Bites, - *By Dr. W. H. Stowe* 147

The Crawford Crown, - - - *By H. H. Johnson* 149

Examination of School Children's Teeth, - - - 151

EDITORIAL, - - - - - - - - - - 155

CORRESPONDENCE, - - - - - - - 156

ANNOUNCEMENTS, - - - - - - - - - 158

INDENTS, - - - - - - - - - 160

